# Effects of bezafibrate on lipid and glucose metabolism in dyslipidemic patients with diabetes: the J-BENEFIT study

**DOI:** 10.1186/1475-2840-11-29

**Published:** 2012-03-23

**Authors:** Tamio Teramoto, Kohji Shirai, Hiroyuki Daida, Nobuhiro Yamada

**Affiliations:** 1 Department of Internal Medicine, Teikyo University School of Medicine, 2-11-1 Kaga, Itabashi-ku, Tokyo, Japan; 2 Department of Internal Medicine, Sakura Medical Center, Toho University School of Medicine, 564-1 Shimoshizu, Sakura-shi, Chiba, Japan; 3 Department of Cardiovascular Medicine, Juntendo University, 2-1-1 Hongo, Bunkyo-ku, Tokyo, Japan; 4 University of Tsukuba, 1-1-1 Tennodai, Tsukuba-shi, Ibaraki, Japan

**Keywords:** Bezafibrate, Diabetes, Triglyceride, HbA1c

## Abstract

**Background:**

The hypoglycemic effect of bezafibrate is well established, but administration to a large population of patients with diabetes has not been reported. We investigated glycemic control, relationship between lipid metabolism and HbA1c, and safety in diabetic patients treated with bezafibrate.

**Methods:**

A prospective, observational analysis was conducted on 6,407 dyslipidemic patients suffering from diabetes or hyperglycemia who had not received bezafibrate previously. Subanalyses were performed on the concomitant use of diabetes drugs, diabetes duration, and baseline HbA1c levels.

**Results:**

Bezafibrate significantly decreased HbA1c irrespective of concomitant use of other diabetes drugs in a baseline-HbA1c-dependent manner, with patients with a shorter diabetes duration showing a greater decrease in HbA1c than those with longer-term disease. The rate of change in triglyceride levels was significantly associated with that in HbA1c. Adverse drug reactions occurred in 306 patients (5.1%), of which reactions in 289 were not severe (94.4%).

**Conclusions:**

Bezafibrate significantly improved HbA1c in patients with diabetes given individualized treatment. Bezafibrate may offer clinicians an improved modality for the amelioration of disease course and improvement of outcome in these patients.

## Background

The prevalence of patients with or at risk of type 2 diabetes is increasing rapidly in Japan, which is in the top 10 of Asian countries with respect to population diagnosed with diabetes and impaired glucose tolerance [1]. Hyperglycemia in patients with type 2 diabetes places them at significant risk for cardiovascular events and other diabetic complications [2], as shown for example by the United Kingdom Prospective Diabetes Study (UKPDS 35), which demonstrated a strong association between the risk of diabetic complications and hyperglycemia [3]. Patients with type 2 diabetes also tend to have higher triglyceride (TG) and lower high-density lipoprotein cholesterol (HDL-C) levels than non-diabetics [4]; for example, Lehto et al. reported that the simultaneous presence of hyperglycemia with either low HDL-C or high TG levels increased the risk of cardiovascular events up to three-fold in patients with type 2 diabetes [5]. Effective treatment of type 2 diabetes must therefore involve the management of blood glucose and lipids, including TG and HDL-C levels.

The ability of bezafibrate to reduce TG, cholesterol, and blood glucose levels in patients with diabetes was first reported over 30 years ago [6,7], and the drug has become widely used for treating dyslipidemia, particularly to improve TG and HDL-C levels [reviewed in [8]]. Moreover, previous research has shown that bezafibrate functions as an agonist of PPAR nuclear transcription factors, which play an important role in glucose and lipid metabolism [9-13]. Although as indicated above, bezafibrate improves lipid and glucose metabolism [14-17], we are unaware of any detailed investigation of its effects in a large cohort of patients with diabetes.

Here, we conducted a 24-week prospective observational study of bezafibrate in the treatment of dyslipidemic patients with diabetes, designated the "Japan BEzafibrate cliNical EFfectIveness and Tolerability (J-BENEFIT)" study.

## Methods

### Subjects

This prospective observational study of dyslipidemic patients with diabetes was conducted as post-marketing surveillance to evaluate the efficacy and safety of bezafibrate therapy. We included only those patients who met all inclusion criteria and did not have any conditions listed in the exclusion criteria. The inclusion criteria were as follows:

1. No prior administration of bezafibrate

2. Serum TG ≥ 1.7 mmol/l (≥ 150 mg/dl) and/or serum total cholesterol (TC) ≥ 5.7 mmol/l (≥ 220 mg/dl)

3. Diagnosis of diabetes or most recent fasting blood glucose (FBG) ≥ 6.1 mmol/l (≥ 110 mg/dl)

The exclusion criteria were as follows:

1. Undergoing dialysis treatment

2. Severe renal disease (on dialysis or in renal failure)

3. Blood creatinine ≥ 152.5 μmol/l (≥ 2.0 mg/dl)

4. History of bezafibrate hypersensitivity

5. Pregnant or possibly pregnant

Subjects were enrolled by centralized registration from June 2003 to March 2005, and the study was conducted from June 2003 to September 2005. Patients were administered 400 mg/day bezafibrate for 24 weeks. We evaluated the efficacy and safety of bezafibrate in each analysis group. Safety was evaluated in all patients whose case report forms were collected, except those with protocol violations or insufficient data for safety analysis. Efficacy was evaluated in all patients except those with protocol noncompliance or insufficient data for efficacy analysis from safety analysis group. Efficacy endpoints included lipid metabolism parameters such as TG, TC, low-density cholesterol (LDL-C), HDL-C, non-HDL-C, and TG/HDL ratio and glucose metabolism parameters such as FBG, HbA1c, and homeostasis model assessment insulin resistance (HOMA-R). Laboratory tests were performed at each of the 1,066 institutions. Efficacy endpoints were assessed at baseline and after 24 weeks of treatment. For observations < 24 weeks, efficacy endpoints were assessed at the last measurement (12 weeks treatment onward). LDL-C was calculated by the Friedwald formula [18] in patients with TG < 4.4 mmol/l. The TG/HDL-C ratio and non-HDL-C were calculated using TC, TG, and HDL-C values. HbA1c was measured according to the Japan Diabetes Society's (JDS) method [19] and then converted to the corresponding National Glycohemoglobin Standardization Program (NGSP) value. HbA1c value was estimated as an NGSP-equivalent value calculated with the following formula:

$$\text{HbA}1\text{c }\left(\text{NGSP}\right)\;=\text{ HbA}1\text{c }\left(\text{JDS}\right)\;+\;0.4$$

considering the relational expression of HbA1c (JDS) measured with the previous Japanese standard substance and methods and HbA1c (NGSP) [19]. Safety endpoints were evaluated as the incidence of adverse drug reaction (ADR), and included laboratory value abnormalities for which an association with bezafibrate could not be ruled out.

### Statistical analysis

All statistical analyses were performed using SAS version 8.12 (SAS Institute). A paired *t*-test was used to compare endpoints after treatment versus baseline with two-tailed significance set at 5%. ANOVA was performed to evaluate linear trends among subgroups classified by baseline HbA1c levels and duration of diabetes. Univariate and stepwise multivariate regression analyses were performed to evaluate independent predictors of the change in HbA1c. The explanatory variables examined were patient age, sex, baseline BMI, baseline HbA1c, administration of diabetes drugs during the study, duration of diabetes, and rate of change of each lipid including TC, LDL-C, TG, and HDL-C. This study was conducted in compliance with good post-marketing surveillance practice and the guidelines on the methods for surveillance of the results of prescription drug use issued by the Ministry of Health, Labour, and Welfare, Japan.

## Results

### Patients

We enrolled 6,449 patients and collected 6,407 case report forms. Safety was evaluated in 5,978 patients. Patients were excluded from safety analysis for the following reasons, namely 1) protocol violation: some patients did not receive bezafibrate (n = 13), had a delay in enrollment (n = 292), and had a history of bezafibrate treatment (n = 20); and 2) insufficient data for safety analysis (n = 104). A total of 429 of 6407 patients were excluded. Patients were excluded from efficacy analysis for the following reasons: 1) protocol violation: patients had neither TG ≥ 1.7 mmol/l nor TC ≥ 5.7 mmol/l (n = 920), or patients had neither diabetes nor FBG ≥ 6.1 mmol/l (n = 653, overlapping); 2) insufficient data for efficacy analysis: dose or duration of bezafibrate unknown (n = 86); data incomplete for baseline or after administration for any one of TC, TG, and HbA1c (n = 2,387); pretreatment for hyperlipidemia unknown or pretreatment with diabetes drugs, or both (n = 783); unknown concomitant drug for treating hyperlipidemia or unknown diabetes drug (n = 6); or both (these cases overlapped, resulting in 2,662 cases being excluded from safety analyses).

Patients' backgrounds in the efficacy analysis group are shown in Table 1. The number of patients with diabetes was 3,312 (99.9%), of whom 3,235 (97.6%) were diagnosed with type 2 diabetes. Mean duration of diabetes was 6.6 ± 5.9 years. A total of 602 (18.2%) and 1,977 (59.6%) patients had been previously treated for dyslipidemia and diabetes, respectively. Medication characteristics of the efficacy analysis group showed that while most patients (3,149; 95.0%) were treated for dyslipidemia with bezafibrate alone, 167 patients (5.0%) took other lipid-lowering drugs concurrently (Table 2).

**Table 1:** Characteristics of patients in the efficacy analysis group

Characteristics of Patients	n (%) or mean ± SD
Number	3316

Entry category	

Type 1 diabetes, n/%	40 (1.2%)

Type 2 diabetes, n/%	3235 (97.6%)

Diabetes (diagnosis unknown), n/%	37 (1.1%)

FBG ≥ 6.1 [mmol/l], n/%	4 (0.1%)

Outpatients, n/%	3212 (96.9%)

Men, n/%	2074 (62.5%)

Age, mean/SD	61.0 ± 12.0

≥65 yeas, n/%	1400 (42.2%)

BMI (kg/m^2^), mean/SD	25.7 ± 3.7

Smoking, n/%	825 (24.9%)

Alcohol consumption, n/%	1129 (34.0%)

Complication, n/%	2397 (72.3%)

Hepatic disease, n/%	339 (10.2%)

Kidney disease, n/%	51 (1.5%)

Hypertension, n/%	1614 (48.7%)

Heart disease, n/%	325 (9.8%)

Duration of hyperlipidemia (years), mean/SD	4.7 ± 4.3

Duration of diabetes (years), mean/SD	6.6 ± 5.9

Previous treatment of hyperlipidemia, n/%	602 (18.2%)

Previous treatment of diabetes, n/%	1977 (59.6%)

Total cholesterol (mmol/l), mean/SD	5.76 ± 1.03

LDL cholesterol (mmol/l), mean/SD	3.19 ± 0.95

HDL cholesterol (mmol/l), mean/SD	1.18 ± 0.33

TG (mmol/l), mean/SD	3.72 ± 2.45

HbA1c (%), mean/SD	7.69 ± 1.52

FBG (mmol/l), mean/SD	9.01 ± 3.49

^a^Standard Deviation

**Table 2:** Drug treatment

	Medication	n (%)
Total number of patients		3316

Concomitant administration of lipid-lowering drug during the study		

No concomitant drug		3149 (95.0%)

Concomitant administration		167 (5.0%)

statin		119 (3.6%)

others		48 (1.4%)

diabetes drug administration before and during the study		

-Before-	-During-	

Not taken	Not taken	818 (24.7%)

Not taken	Taken	521 (15.7%)

Taken	Not taken	106 (3.2%)

Taken	Taken	1871 (56.4%)

No concomitant diabetes drug during the study		924 (27.9%)

Concomitant treatment with diabetes drug during the study		2392 (72.1%)

Single treatment with:		

Sulfonylurea		732 (22.1%)

Glinide		188 (5.7%)

Alpha-glucosidase inhibitor		169 (5.1%)

Biguanide		93 (2.8%)

Thiazolidine		49 (1.5%)

Insulin		156 (4.7%)

Combination therapies:		

Sulfonylurea + alpha-glucosidase inhibitor		229 (6.9%)

Sulfonylurea + biguanide		212 (6.4%)

Sulfonylurea + thiazolidine		91 (2.7%)

Other		473 (14.3%)

All patients were classified into four subgroups according to diabetes drug administration before and during the study as follows: diabetes drug not taken before or during the study (n = 818, 24.7%), taken only during the study (n = 521, 15.7%), taken only before the study (n = 106, 3.2%) and taken before and during the study (n = 1,871, 56.4%). Thus, a diabetes drug was taken by 2,392 patients (72.1%) during the study. The most frequently used diabetes drug regimen was single administration of sulfonylureas (22.1%) followed by combined use of sulfonylureas and an α-glucosidase inhibitor (6.9%) (Table 2). The number of patients receiving both monotherapy and combination use of diabetes drugs was as follows: sulfonylureas (n = 1,505, 45.4%), glinides (n = 341, 10.3%), α-glucosidase inhibitor (n = 685, 20.7%), biguanide (n = 550, 16.6%), thiazolidine (n = 287, 8.7%), and insulin (n = 284, 8.6%) (data not shown). All of 3,316 patients took bezafibrate at least 6 weeks, of whom 3,299 (99.5%) took the drug for at least 12 weeks and 17 (0.5%) for at least six weeks. The dose of bezafibrate was 400 mg/day in 2,728 (82.3%) patients and 200 mg/day in 472 (14.2%).

### Effect of bezafibrate on blood lipid and glucose levels

Table 3 presents the changes in lipid and glucose parameters at baseline and after bezafibrate administration. All lipid values except LDL-C improved significantly compared with baseline values. Significant decreases from baseline were observed for TC, TG, non-HDL-C, and TG/HDL-C ratio; in contrast, a significant increase was observed in HDL-C. Although LDL-C levels decreased significantly in patients with LDL-C ≥ 3.6 mmol/l at baseline (n = 737), they increased significantly in those with LDL-C < 3.6 mmol/l at baseline (n = 1,489). Thus, LDL-C levels increased significantly overall. Moreover, HbA1c, FBG, and HOMA-R levels decreased significantly.

**Table 3:** Analysis of lipid and glycemic parameters

	n	Baseline mean ± SD	After administration mean ± SD	Difference	Percentage
TC (mmol/L)	3316	5.76 ± 1,03	5.45 ± 0.94	-0.31 ± 1.00 §	-5.4%

LDL-C (mmol/L)	2226	3.19 ± 0.95	3.25 ± 0.81	0.05 ± 0.90 ‡	1.6%

TG (mmol/L)	3316	3.72 ± 2.45	2.03 ± 1.50	-1.69 ± 2.08 §	-45.4%

HDL-C (mmol/L)	2818	1.18 ± 0.32	1.34 ± 0.35	0.17 ± 0.25 §	14.0%

non-HDL-C (mmol/L)	2818	4.57 ± 0.99	4.11 ± 0.96	-0.46 ± 0.09 §	-10.1%

TG/HDL	1818	3.49 ± 2.75	1.70 ± 1.59	-1.79 ± 2.31 §	-51.3%

HbA1c (%)	3316	7.69 ± 1.52	7.22 ± 1.28	-0.47 ± 1.21 §	-6.2%

FBG (mmol/L)	2387	9.00 ± 3.46	7.81 ± 2.89	-1.19 ± 3.33 §	-13.2%

HOMA-R	102	4.46 ± 4.91	3.38 ± 3.83	-1.08 ± 4.78 †	-24.3%

"Difference" indicates the value for the change from baseline. "Percentage" indicates rate of change from baseline. A paired *t*-test was used to assess statistically significant differences from baseline for each group. Symbols for p values in this and the tables that follow: †: p < 0.05, ‡: p < 0.01, §: p < 0.001

### Effect of bezafibrate on blood glucose levels - subgroup analysis

Table 4 summarizes the changes in HbA1c concentrations after treatment, classified according to use of diabetes drug and baseline HbA1c levels. We analyzed 2,086 patients whose baseline HbA1c values were ≥7.0%. HbA1c levels decreased significantly by -0.76% from baseline in all patients. This change was greater in patients whose baseline HbA1c levels were higher. The trend analysis showed significant differences between the HbA1c-subgroups. Similar results were observed in all medication subgroups classified by diabetes drug administration before and during the study.

**Table 4:** HbA1c levels as a function of concomitant diabetes drug use and baseline HbA1c levels

Medication classification	HbA1c classification	n	Baseline mean ± SD	After administration mean ± SD	Difference	
All patients	Total	2086	8.46 ± 1.40	7.71 ± 1.31	-0.76 ± 1.38 §	
	7-8%	1010	7.46 ± 0.29	7.20 ± 0.77	-0.25 ± 0.74 §	p < 0.001
	8-9%	555	8.44 ± 0.28	7.75 ± 1.02	-0.69 ± 1.01 §	
	≥9%	521	10.45 ± 1.29	8.64 ± 1.79	-1.81 ± 1.97 §	

Diabetes drug use	Subtotal	254	7.84 ± 1.04	7.28 ± 1.00	-0.56 ± 1.06 §	
Before study: not taken &	7-8%	186	7.38 ± 0.26	7.06 ± 0.67	-0.32 ± 0.66	p < 0.001
During study: not taken	8-9%	46	8.38 ± 0.26	7.64 ± 1.08	-0.74 ± 1,03 §	
	≥9%	22	10.64 ± 1.24	8.48 ± 1.85	-2.16 ± 2.03 §	

	Subtotal	397	8.89 ± 1.66	7.42 ± 1.36	-1.47 ± 1.88 §	
Before study: not taken &	7-8%	147	7.46 ± 0.29	7.06 ± 0.85	-0.39 ± 0.86 §	p < 0.001
During study: taken	8-9%	109	8.45 ± 0.29	7.31 ± 0.96	-1.15 ± 0.97 §	
	≥9%	141	10.73 ± 1.40	7.89 ± 1.84	-2.84 ± 2.30 §	

	Subtotal	59	8.26 ± 1.33	7.79 ± 1.23	-0.47 ± 1.10 ‡	
Before study: taken &	7-8%	34	7.48 ± 0.28	7.26 ± 0.83	-0.22 ± 0.85	p = 0.003
During study: not taken	8-9%	14	8.50 ± 0.28	8.09 ± 1.03	-0.41 ± 0.93	
	≥9%	11	10.38 ± 1.67	9.05 ± 1.51	-1.32 ± 1.59 †	

	Subtotal	1376	8.46 ± 1.33	7.86 ± 1.31	-0.60 ± 1.20 §	
Before study: taken &	7-8%	643	7.48 ± 0.29	7.28 ± 0.76	-0.20 ± 0.73 §	p < 0.001
During study: taken	8-9%	386	8.44 ± 0.27	7.88 ± 1.00	-0.56 ± 0.98 §	
	≥9%	347	10.32 ± 1.22	8.94 ± 1.69	-1.38 ± 1.66 §	

Baseline HbA1c levels for 2086 patients were ≥ 7.0%. A paired *t*-test was used to assess statistically significant differences from baseline for each group. P values were determined by ANOVA to assess statistically differences from baseline among baseline HbA1c-subgroup

There were 1,464 patients for which we could determine the change in HbA1c levels as a function of duration of diabetes (Table 5). HbA1c levels significantly decreased in all diabetes-duration subgroups, most strongly in the subgroup with diabetes for < 1 year. Trend analysis showed significant differences between the diabetes-duration subgroups. Similar results were also observed in the medication subgroup of patients who were not administrated diabetes drug before or during the study.

**Table 5:** HbA1c-levels as a function of the duration of diabetes

Medication classification	Duration of diabetes classification	n	Baseline maen ± SD	After administration mean ± SD	Difference mean ± SD	
All cases	Total	1464	8.44 ± 1.34	7.76 ± 1.29	-0.68 ± 1.34 §	
	< 1 year	70	8.65 ± 1.63	7.04 ± 1.08	-1.60 ± 1.82 §	p = 0.002
	1-5 years	503	8.38 ± 1.35	7.69 ± 1.38	-0.69 ± 1.50 §	
	5-10 years	432	8.37 ± 1.25	7.81 ± 1.22	-0.56 ± 1.02 §	
	≥ 10 years	459	8.56 ± 1.36	7.90 ± 1.25	-0.66 ± 1.27 §	

Diabetes drug use	Subtotal	147	7.81 ± 0.95	7.33 ± 1.05	-0.47 ± 1.00 §	
Before study: not taken &	< 1 year	14	7.72 ± 0.89	6.52 ± 0.75	-1.20 ± 1.31 ‡	p = 0.012
During study: not taken	1-5 years	75	7.73 ± 0.78	7.45 ± 1.08	-0.28 ± 0.88 ‡	
	5-10 years	38	7.85 ± 1.15	7.38 ± 1.13	-0.47 ± 0.97 ‡	
	≥ 10 years	20	8.06 ± 1.16	7.36 ± 0.76	-0.70 ± 1,04 ‡	

Baseline HBA1c levels for 1464 patients were ≥ 7.0%. P values were determined by ANOVA to assess statistically difference among duration of diabetes-subgroup

We also analyzed HbA1c levels according to concomitant use of a single diabetes drug during the study. Changes in HbA1c levels for each drug were as follows: sulfonylureas, n = 536, -0.84%, p < 0.001; glinides, n = 125, -0.82%, p < 0.001; alpha glucosidase inhibitor, n = 70, -0.66%, p < 0.001; biguanide, n = 239, -0.73%, p < 0.001; thiazolidine, n = 28, -0.64%, p = 0.004; insulin: n = 130, -0.59%, p < 0.001. Each regimen showed a significant decrease in HbA1c. There were no significant differences between any of the regimens (p = 0.363) (data not shown).

### Factors influencing HbA1c levels

We conducted univariate and multivariate regression analyses to evaluate the influence of bezafibrate on HbA1c levels. For initial univariate analysis of 1,854 patients, the difference in HbA1c levels was as an objective variable. Explanatory variables were patient age, sex, baseline BMI, baseline HbA1c level, diabetes-drug administration during the study, duration of diabetes, and rate of change in levels of each lipid (TC, LDL-C, TG, and HDL-C). Based on these results, baseline BMI, baseline HbA1c, diabetes-drug administration, rate of change in TG, HDL-C, and TC were found to be significant variables.

**Figure 1:**
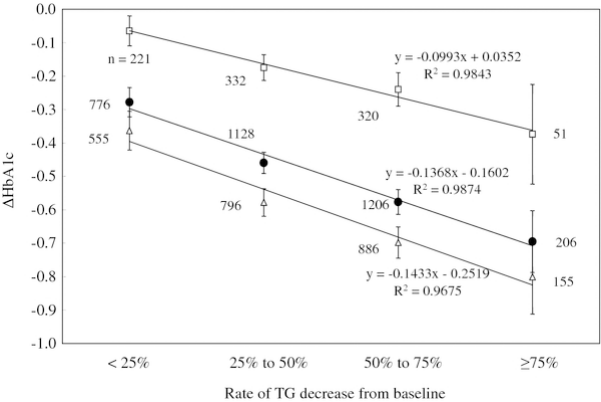
**TG and HbA1c levels in patients depending on use of a diabetes drug**. The graphs show the changes in HbA1c levels determined for all patients (•), patients taking a diabetes drug (**Δ**), and patients not taking a diabetes drug (☐).

We next performed multivariate regression analysis using these variables and found that the estimated influence rate per unit change in each variable on HbA1c was as follows: baseline HbA1c, -0.489; baseline BMI, 0.016; diabetes-drug administration, 0.176; rate of change in TG, 0.004; and rate of change in TC, 0.005. The rate of change in HDL-C was not a significant variable (Table 6). Further, the average rates of change in TG and TC for all subjects at 24 weeks were -45.4% and -5.4%, respectively (Table 3). When the rate of change in each lipid was considered, their estimated influences on altering HbA1c levels were -0.182% and -0.027%, respectively. Next, we analyzed the relationship between rate of change in TG and amount of change in HbA1c in 3,316 patients (Figure 1). Irrespective of diabetes drug administration during the study, a strong positive relationship was observed between the rates of change in TG and amount of change in HbA1c levels.

**Table 6:** Stepwise multiple regression analysis of the change in HbA1c levels in relation to explanatory variables

Explanatory variable	Regression coefficient	SE	*t-*value	p-value
Baseline HbA1c (%)	-0.489	0.015	-31.79	< 0.001
Baseline BMI (kg/m^2^)	0.016	0.006	2.70	0.007
Diabetes drug administration	0.176	0.049	3.59	< 0.001
Change rate in TG (%)	0.004	0.001	6.34	< 0.001
Change rate in TC (%)	0.005	0.001	4.13	< 0.001
Change rate in HDL-C (%)	-0.002	0.001	-1.72	0.085

R^2 ^= 0.3884. Each estimated value indicates the influence in HbA1c per 1 unit change in each variable. The estimated values of diabetes-drug administration indicate the rates of influence in patients with and without drug

### Safety

Drug safety was assessed for 5,978 patients. ADRs were experienced by 306 patients (5.1%) (Table 7). The most common ADRs were increased blood creatine phosphokinase (0.8%), blood creatinine (0.8%), blood urea (0.5%), renal impairment (0.3%), and aspartate aminotransferase (0.3%). Severe ADRs were experienced by 17 patients (0.28%) and mainly included stroke (n = 2, 0.03%), renal impairment (n = 2, 0.03%), elevated blood urea nitrogen (n = 2, 0.03%), abnormal hepatic function (n = 1, 0.02%), pneumonia (n = 1, 0.02%), rhabdomyolysis (n = 1, 0.02%), hypoglycemia (n = 1, 0.02%), and others. ADR rate increased slightly in patients concurrently taking diabetes drugs (4.3% not taking vs. 5.6% taking), but no apparent clinical differences were observed. One hypoglycemia case occurred in a patient concurrently taking a diabetes drug. Rhabdomyolysis was observed in five patients, four of whom were treated with a diabetes drug. No ADRs were observed that could be unequivocally ascribed to concurrent diabetes drug use.

**Table 7:** Adverse drug reactions in the safety analysis set

Adverse drug reactions	Cases	Cases without diabetes drug	Cases with diabetes drug
Number	5978	2382	3596
ADRs	306 (5.1%)	103 (4.3%)	203 (5.6%)
Blood creatine phosphokinase increased	48 (0.8%)	17 (0.7%)	31 (0.9%)
Blood creatinine increased	46 (0.8%)	18 (0.8%)	28 (0.8%)
Blood urea increased	29 (0.5%)	10 (0.4%)	19 (0.5%)
Renal impairment	19 (0.3%)	8 (0.3%)	11 (0.3%)
Aspartate aminotransferase increased	18 (0.3%)	7 (0.3%)	11 (0.3%)
Myalgia	17 (0.3%)	6 (0.3%)	11 (0.3%)
Hepatic function abnormal	15 (0.3%)	2 (0.1%)	13 (0.4%)
Pruritus	13 (0.2%)	6 (0.3%)	7 (0.2%)
Alanine aminotransferase increased	13 (0.2%)	5 (0.2%)	8 (0.2%)
Rash	12 (0.2%)	6 (0.3%)	6 (0.2%)
Dyspepsia	7 (0.1%)	1 (0.0%)	6 (0.2%)
Others	72 (1.2%)	19 (0.8%)	53 (1.5%)

## Discussion

In this study, we showed that treatment with bezafibrate significantly reduced HbA1c levels in patients with diabetes complicated by dyslipidemia. This effect was observed in a baseline HbA1c-dependent manner, regardless of concurrent use of diabetes drugs. Patients with a shorter duration of diabetes showed greater decreases in HbA1c levels. Further, the rate of change in triglyceride levels was related to changes in HbA1c levels. Adverse drug reactions occurred in 306 patients (5.1%), but these were not severe in 289 (94.4%). Taken together, these results suggest that bezafibrate, which improves both lipid and blood glucose metabolism, should be considered suitable for the treatment of dyslipidemia in patients with diabetes.

The importance of the rigorous control of glycemia in diabetes is confirmed by the striking findings that a 1% reduction in HbA1c level is associated with a 21% decrease in risk of death related to diabetes [3], and that a 1-mmol/l increase in TG level is associated with a 32% increased risk of a cardiovascular event [20]. HbA1c correlates significantly with both TG and HDL-C in patients with type 2 diabetes [21,22]. Further, patients with type 2 diabetes tend to have higher TG and lower HDL-C values than dyslipidemic patients without type 2 diabetes [4]. TG levels are significantly higher and HDL-C levels significantly lower in patients with relatively poor glycemic control than in those with adequate control [21]. These findings confirm the importance of controlling both TG and HDL-C, in addition to blood glucose, in the treatment of type 2 diabetes.

Statins have been widely used to treat dyslipidemia, and evidence supports their role in preventing cardiovascular events [23-26]. However, fibrates, including bezafibrate, generally have a stronger effect on TG and HDL-C than statins. Atorvastatin 20 mg/day and rosuvastatin 10 mg/day reduced TG levels by 4.6% and 36.4%, and increased or decreased HDL-C levels by 4.5% and 2.0%, respectively [27]. Although rosuvastatin (5-20 mg/day) decreased TG levels by 29%-31% and increased HDL-C levels by 12.4%-16.7%, bezafibrate (400 mg/day) decreased TG levels by 45% and increased HDL-C levels by 43% [28]. The present study also revealed that bezafibrate decreased the rate of change in TG level by 45.4% and the rate of increase in HDL-C level by 14.0% (Table 3). These results suggest that the effect of fibrates on TG and HDL-C levels is stronger than that of statins.

Although controversial, evidence suggests that fibrates have a lower association with cardiovascular events than statins. Indeed, a recent meta-analysis of 18 fibrate trials [29] revealed that fibrates significantly reduce the risk of cardiovascular events, but not uniformly [30-34]. In the ACCORD study, cardiovascular disease risk in 5,581 patients [30] receiving combination therapy with a statin plus a fenofibrate was no different than that in patients with statin monotherapy. Further, in the BIP study of 3,090 patients [32], bezafibrate did not significantly reduce the primary endpoint (fatal or nonfatal myocardial infarction or sudden death).

Common findings between these studies [30-33] are as follows: first, baseline TG values were not high, averaging 1.8 mmol/l in ACCORD and 1.6 mmol/l in BIP. Second, subgroup analysis of patients with baseline TG values ≥ 2.2 mmol/l exhibited potent (31%) risk reduction in ACCORD and 39.5% (*P *= 0.02) in BIP. Results of the FIELD study were similar [34]. We therefore conclude that fibrates can play a prominent role in reducing the risk of cardiovascular events in patients with high TG levels. Further, recent reports indicate that statin therapy is associated with a slightly higher risk of developing diabetes [35,36]. These findings suggest that if a patient is at low risk for cardiovascular disease, the potential for statins to increase diabetes risk should be taken into account.

Bezafibrate reduces blood glucose levels in patients with type 2 diabetes [14,15]. Elkeles et al. reported that bezafibrate significantly decreased the combined incidence of ischemic change in ECG and documented myocardial infarction in patients with type 2 diabetes [37]. Further, Tenenbaum et al. reported that bezafibrate decreased the development and delayed the onset of type 2 diabetes in patients with impaired fasting glucose [38]. Therefore, bezafibrate may be more suitable than statins for treating dyslipidemic patients with type 2 diabetes if their cardiovascular risk is low. To our knowledge, our present study is the first to demonstrate the beneficial effects of bezafibrate on lipid and glucose metabolism in a large number of patients with diabetes.

Consistent with studies cited above, we showed here that bezafibrate improved blood glucose and lipid levels. All lipid values except LDL-C improved significantly from baseline, with TG, HDL-C levels and the TG/HDL ratio in particular showing significant changes. Further, HbA1c, FBG, and HOMA-R improved significantly from baseline. HbA1c levels decreased by -0.47% in all patients and by -0.76% in patients with baseline HbA1c levels ≥7.0%. Notably, bezafibrate showed a potent hypoglycemic effect regardless of concurrent diabetes drug use. On analysis stratified by diabetes duration, bezafibrate alone reduced HbA1c in all subgroups, most notably in those with diabetes for < 1 year. Therefore, bezafibrate monotherapy may be appropriate for treating early-onset type 2 diabetes coexisting with hypertriglyceridemia. Alternatively, hyperglycemic patients with inadequate blood glucose control might benefit from concomitant administration of bezafibrate and a diabetes drug.

We also demonstrated here that as TG levels decreased, those of HbA1c decreased by 0.47% in all patients (Figure 1 and Table 3). Ogawa reported that bezafibrate decreased TG levels by 50% and decreased HbA1c levels from 7.2% to 6.9% in patients with type 2 diabetes [16]. Taniguchi reported similar results [15]. In contrast, the three-year SENDCAP study of 164 patients with type 2 diabetes reported that while bezafibrate decreased TG significantly, the change in HbA1c levels over the course of the study was not significant between bezafibrate and the control groups [37]. Although long-term studies will be required to confirm this observation, average baseline TG levels were lower than those cited above, leading us to conclude that this may have caused the change in HbA1c levels.

Bays et al. reported the following two relationships between fatty acid levels and type 2 diabetes [39]: (1) chronically increased plasma free-fatty acid induces hepatic and muscle insulin resistance and impairs insulin secretion; and (2) enlarged fat cells become insulin-resistant, with diminished capacity to store fat. When the storage capacity in adipocyte tissue is exceeded, lipids flow over into muscle and liver, causing muscle and hepatic insulin resistance. Bezafibrate has been reported to increase fatty acid degradation via beta-oxidation in the peroxisomes and mitochondria [40-42]. Further, Van der Ziji et al. reported that pancreatic fatty acid accumulation is related to β cell dysfunction [43], and Fernandes-Santos et al. reported that bezafibrate prevented pancreatic fat accumulation and hypertrophy in mice while attenuating glucose intolerance and insulin resistance [44]. These studies suggest that plasma free fatty acid and ectopic fat accumulation involve glucose tolerance, and that bezafibrate improves glucose tolerance through fatty acid degradation via activating beta-oxidation.

ADRs were observed in 306 (5.1%) of 5,978 patients in the safety analysis group. The most frequent were increases in blood levels of creatine phosphokinase (0.8%), creatinine (0.8%) and urea (0.5%). No specific differences were observed related to the use or non-use of diabetes drugs. Betteridge and O' Bryan-Tear [45] and Beggs et al. [46] reported, respectively, that 7.7% (7/91) and 5.4% (7/130) of ADRs were caused by bezafibrate administration. The rate of ADRs in the present study is comparable to their result.

In the present study, concurrent use of the biguanide drug metformin was 16.6%. The guidelines of the American Diabetes Association and the European Association for the Study of Diabetes recommend initial treatment of type 2 diabetes mellitus with metformin [47]. Since 2009, metformin treatment has been restricted to cases that do not respond to sulfonylureas. The maximum dose was 750 mg/day, which, until 2010 in Japan at least, is much less than the dose administered in Western countries. Therefore, the results of clinical trials in Western countries cannot be directly applied to Japanese patients. Further, the majority of Japanese patients with type 2 diabetes mellitus are less obese and less insulin-resistant than European and American patients [48]. Currently in Japan, the maximum dose of metformin is 2,250 mg/day.

Our study has several important limitations. First, it was conducted under a prospective observational cohort design with no control arm, and it was not possible to eliminate all confounding factors. Interpretation of our findings, therefore, requires caution until further studies with controls can be conducted. Second, changes in body weight, modification of lifestyle, and use of diabetes drugs were not recorded, and the effects of these variables on glucose metabolism cannot be ruled out. It is, however, important to note that other studies [15,16] reported result similar to ours presented here. Moreover, of the 254 patients not treated with diabetes drugs in the present study, average HbA1c levels decreased by 0.56% after administration of bezafibrate (Table 4), strongly suggesting that bezafibrate alone lowers blood glucose levels. Third, for logistic, economic and other reasons, laboratory measurements could only be performed at each of the 1,066 institutions and not in a central laboratory. Although the JDS, in collaboration with the Japan Society of Clinical Chemistry, developed a national standardization scheme for determining HbA1c levels in 1995 in Japan [49], and Nihei et al. reported that commutability among the most frequently used analytical techniques in Japan was secured at a specific level [50], differences in HbA1c levels determined by different methods and laboratories cannot be ruled out. Nevertheless, we believe that the present results demonstrate convincingly the beneficial effects of bezafibrate in patients with type 2 diabetes in clinical settings.

## Conclusions

This 24-week prospective observational study of dyslipidemic patients with diabetes or hyperglycemia showed that bezafibrate significantly reduced HbA1c levels as a function of baseline HbA1c level regardless of concurrent use of diabetes drugs. Further, a correlation was apparent between the rates of change of triglyceride and HbA1c levels. Patients with type 2 diabetes tended to have higher TG and lower HDL-C values [4]. We therefore conclude that the control of both TG and blood glucose levels is an important consideration in the treatment of dyslipidemia complicated by diabetes and that physicians should consider that bezafibrate is an effective therapy in these patients.

## Abbreviations

ADR: Adverse drug reaction; JDS: Japan Diabetes Society; FBG: Fasting blood glucose; HbA1c: Glycated hemoglobin; HDL-C: High-density lipoprotein cholesterol; HOMA-R: Homeostasis model assessment insulin resistance; LDL-C: Low-density lipoprotein cholesterol; NGSP: National Glycohemoglobin Standardization Program; PPAR: Peroxisome proliferator-activated receptor; TC: Total cholesterol; TG: Triglyceride.

## Competing interests

This work was sponsored by KISSEI Pharmaceutical Company, Japan. TT is currently an active member of the GlaxoSmithKline KK scientific advisory board. TT has received research grants from Daiichi-Sankyo Co. Ltd., Astellas Pharma Inc., Kowa Co. Ltd., Shionogi Co. Ltd., Bayer Yakuhin Ltd. and Kissei Pharmaceutical Co. Ltd. KS has received research grants from Daichi Sankyo Co. Ltd., Astellas Pharma Inc., Ohtsuka Pharmaceutical Company, Kowa Co. Ltd., Kissei Pharmaceutical Co. Ltd., Banyu Pharmaceutical Co. Ltd. and Fukuda Denshi Co. Ltd. and has received honoraria as a lecturer from Kowa Co. Ltd., Daiichi Sankyo Co. Ltd., Sunny Health Co. Ltd., Takeda Pharmaceutical Co. Ltd., Fukuda Densi Co. Ltd., and Shionogi Pharmaceutical Co. Ltd. HD is an advisory member of Kowa Co. Ltd. and Sanofi-Aventis KK and has received honoraria for lectures and research grants from Kowa Co. Ltd., Pfizer Inc., Daiichi Sankyo Co. Ltd., AstraZeneca PLC, Astellas Pharma Inc., The Boston Scientific Corporation, Sanofi-aventis KK, Mochida Pharmaceutical Co. Ltd., Takeda Pharmaceutical Co. Ltd. and Dainippon Sumitomo Pharma Co. Ltd.

## Authors' contributions

KS participated in drafting the manuscript. HD performed the statistical analysis. NY participated in the design of the study. TT conceived of the study and participated in its design and coordination and helped draft the manuscript. All authors read and approved the final manuscript.
